# A LATENT VARIABLE APPROACH TO ASSESS DEMENTIA IN MEXICO AND THE UNITED STATES: MEASUREMENT INVARIANCE AND VALIDATION

**DOI:** 10.1093/geroni/igad104.2647

**Published:** 2023-12-21

**Authors:** Joseph Saenz, Christopher Beam, Silvia Mejía-Arango

**Affiliations:** Arizona State University, Phoenix, Arizona, United States; University of Southern California, Los Angeles, California, United States; University of Texas Rio Grande Valley, Edinburg, Texas, United States

## Abstract

Latent dementia indices (LDI) use cognitive and functional data to approximate dementia. Few evaluate the LDI’s utility in cross-national work. This study tests metric measurement invariance of an LDI in the United States and Mexico and evaluates its validity in Mexico. Data included the United States Harmonized Cognitive Assessment Protocol (HCAP, n=3,267), MexCog (n=2,042), and a Mexican clinical validation sample with diagnosed cognitive status (51 cognitively normal, 49 mild cognitive impairment (MCI), and 50 dementia) who received the MexCog battery. The LDI was measured using highly comparable items (13 cognitive and 10 functional). We tested metric measurement invariance of the LDI in MexCog/HCAP samples and evaluated validity by regressing cognitive status on LDI factor scores in the Mexican validation sample using multinomial regression and evaluating how model-implied cognitive status aligned with diagnosis. Full metric invariance of the LDI in the MexCog and HCAP was supported, suggesting that the LDI may be used in cross-national work to validly compare dementia risk factors in the studies. In the Mexican validation sample, LDI factor scores significantly predicted MCI (β=-2.62, p< 0.001) and dementia (β=-5.93, p< 0.001) versus cognitively normal in multinomial regressions. LDI model-implied cognitive status showed moderately high dementia (84.0%) and MCI (88.9%) sensitivity and moderately high dementia (92.0%) and MCI (88.2%) specificity. The LDI may be used to cross-nationally compare risk/protective factors for dementia in MexCog/HCAP studies. Alignment between the LDI and diagnosed cognitive status in the Mexican validation sample supports its validity as a proxy for dementia in Mexico.

